# The use of bipolar coagulation forceps prevented salivary fistula in patients with parotidectomy: a retrospective study

**DOI:** 10.1186/s12903-021-01750-6

**Published:** 2021-08-06

**Authors:** Kun Wu, Keke Zhu, Yingxi Ye, Sainan Li, Hanjiang Wu, Sheng Zhang

**Affiliations:** 1 Department of Stomatology, Second Xiangya Hospital of Central South University, Renmin Road, No. 139, Changsha, 410011 Hunan China; 2 Department of Stomatology, The First Affiliated Hospital of Hunan University of Chinese Medicine, Changsha, Hunan China

**Keywords:** Parotid tumor, Salivary fistulas, Parotidectomy, Bipolar coagulation forceps

## Abstract

**Background:**

Salivary fistula is a relatively common complication in patients who have undergone a parotidectomy. The purpose of this study was to investigate the effects of bipolar coagulation forceps use on salivary fistulas.

**Methods:**

From March 2015 to June 2020, 177 patients who underwent a parotidectomy in the Department of Oral and Maxillofacial Surgery at the Second Xiangya Hospital of Central South University were recruited. The patients were divided into an experimental group and a control group based on whether bipolar coagulation forceps or sutures were used, respectively.

**Results:**

The drainage output of the experimental group was significantly lower than that of the control group (*p* = 0.04). The duration of dressing pressure applied in the experimental group was significantly shorter than that in the control group (*p* = 0.0003). Moreover, the incidence of salivary fistula in the experimental group (9.8%, 8/82) was notably lower than that in the control group (34.7%, 33/95) (*p* < 0.0001). In the logistic regression model for salivary fistula development, both the use of bipolar coagulation forceps (*p* = 0.0021) and drainage output (*p* = 0.0237) were associated with the presence of salivary fistulas.

**Conclusions:**

Our findings indicate that the use of bipolar coagulation forceps decreases the incidence of salivary fistula in patients who have undergone a parotidectomy. The use of bipolar coagulation forceps is a safe, effective, and convenient method to prevent salivary fistulas in patients who undergo a parotidectomy.

*Trial registration*: Current Controlled Trials ChiCTR2100044722, Date: 26/03/2021, Retrospectively registered.

## Background

Salivary gland tumors are relatively rare, constituting approximately 3–4% of all head and neck tumors [1]. Moreover, parotid tumors are the most common type of salivary gland tumors, accounting for 80% of all salivary gland neoplasms [2]. Although most tumors in the minor salivary glands are malignant, the majority of parotid tumors are benign [1]. The manifestations of parotid neoplasms vary widely in pathological diagnoses [3], and the complications of treatment are similar because the tumors are located in the same anatomical region. Surgical treatment is a universally accepted therapy for benign parotid neoplasms. A parotidectomy inevitably destroys the entire parotid gland, leading to various complications, including facial nerve paralysis, salivary fistula, and Frey’s syndrome.

A major concern in surgical procedures is the prevention of bleeding. Bipolar coagulation forceps act as hemostatic devices that control the bleeding during surgical procedures [4]. The passage of an electric current through the tissue produces heating [5], in which the heat production and electric current can be maximized in the straight path between the electrodes [6]. Hot cautery was used to achieve hemostasis by producing a large tissue coagulum, which usually prevents bleeding [6].

Salivary fistula is a relatively common complication that occurs in 5–39% of patients after a parotidectomy [7] which decreasing their quality of life. This complication can also cause visible scarring and wound infections. To prevent these complications, continuous pressure dressing of the parotid region is necessary but may lead to cosmetic issues, prolonged hospitalization, increased costs, and emotional instability [8]. Thus, it is important to identify novel treatments that decrease the occurrence of salivary fistulas.

In the present study, we aimed to investigate the effects of bipolar coagulation forceps use on salivary fistulas.

## Methods

### Patients and study design

**Fig. 1:**
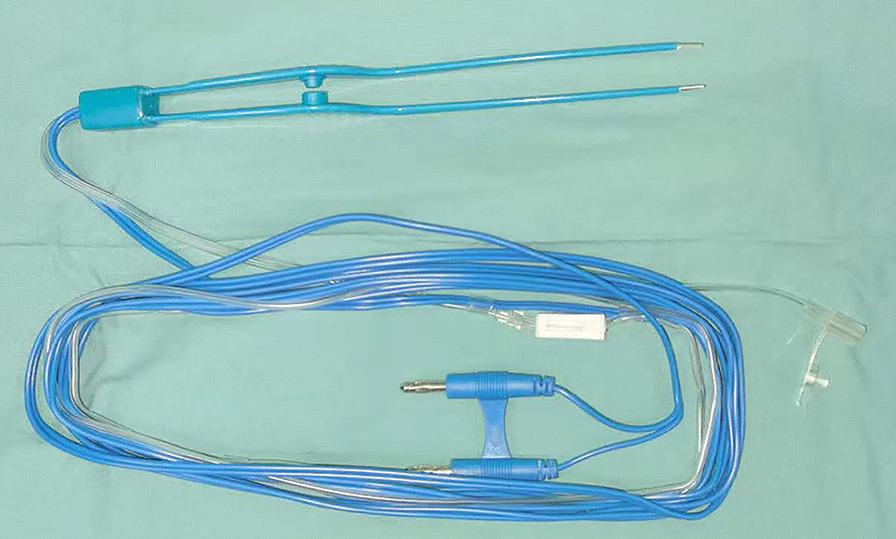
The photograph of bipolar coagulation forceps

**Fig. 2:**
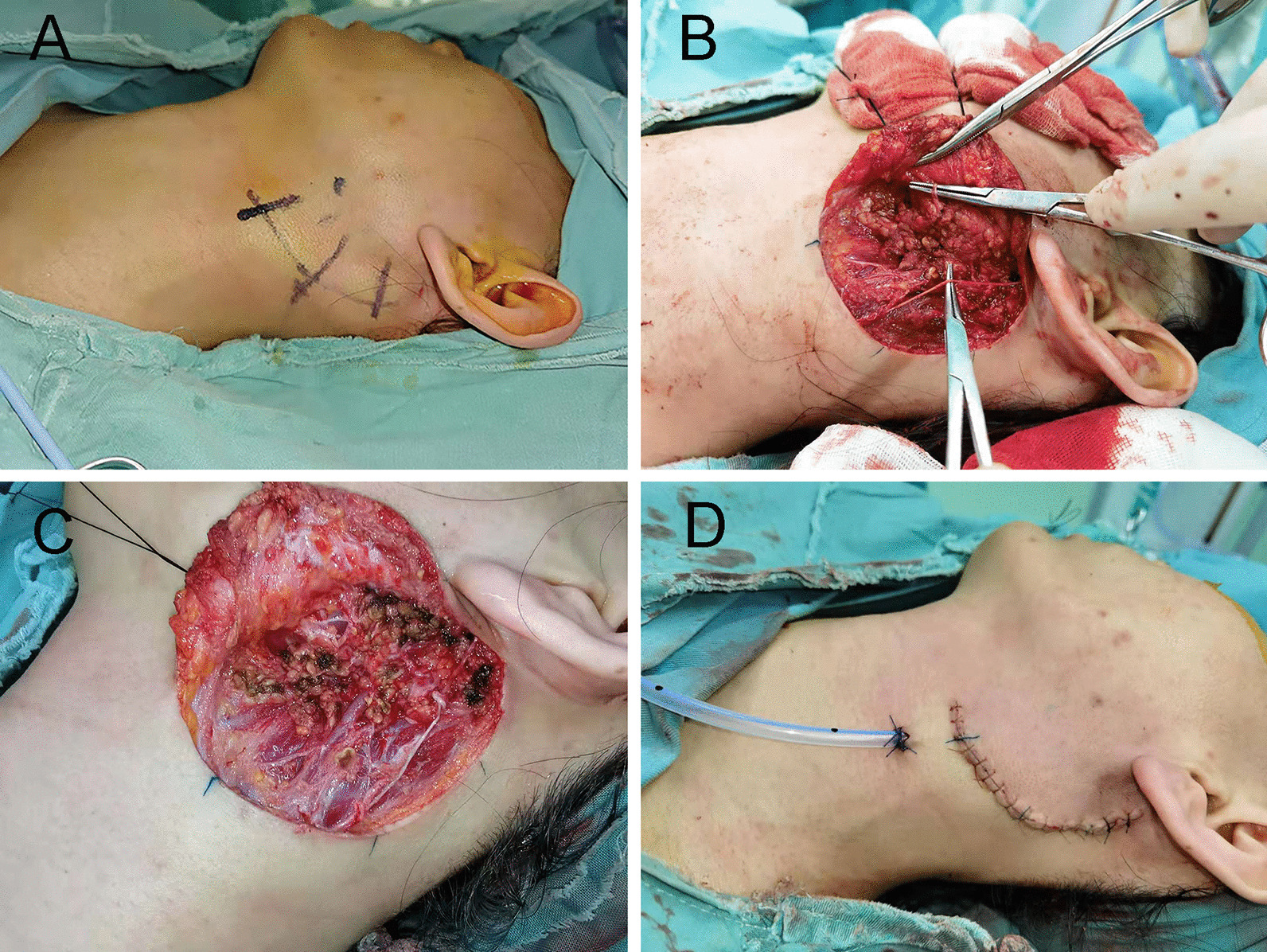
The parotidectomy was performed with bipolar coagulation forceps, **A** surgical incision, **B** partial superficial parotidectomy with a branched facial nerve dissection, **C** the exposed parotid parenchyma treated with bipolar coagulation forceps, **D** suture of surgical wound

This was a retrospective cohort study. From March 2015 to June 2020, 177 patients who had undergone a parotidectomy in the Department of Oral and Maxillofacial Surgery at the Second Xiangya Hospital of Central South University in China were recruited. The exposed parenchyma after dissection of the facial nerve was managed with sutures in the control group and treated with bipolar coagulation forceps (Fig. 1) (TIANEN TECHNOLOGY Co., Ltd., China) in the experimental group (Fig. 2).The study was approved by the institutional review board of the Second Xiangya Hospital, and informed consent was obtained from all participants. Patients with a history of a previous parotidectomy or radiation therapy in the head and neck were excluded. Patients who had been lost to follow-up were excluded. All patients were aged over 18 years.

The surgical procedures were performed by two senior surgeons. The surgical procedures included a tumor and partial superficial parotidectomy with a branched facial nerve dissection, a tumor and partial superficial parotidectomy with a main trunk facial nerve dissection, and a tumor and total parotidectomy with a main trunk facial nerve dissection [3]. Finally, negative pressure drainage devices (B. B. raun Melsungen AG Co., Ltd., Germany) were used to collect postoperative secretions. A restraining bandage was used to maintain continuous pressure on the operative region postoperatively. The 24 h drainage volume was recorded each day until the drainage volume was < 10 mL for two consecutive days or eight days had passed, and the negative pressure drainage tube was removed [3]. All patients underwent routine observation and strict follow-up schedules. The patients were asked to see the doctor once a month during the first postoperative year. Subsequent follow-up visits were performed once every three months. Follow-up management was performed by a single doctor.

Salivary fistula was defined when an effusion developed in surgical region after removing drainage device. Salivary fistula was the only study variable in this study. The application of bipolar coagulation forceps was an exposed factor. The confounding factors included age, sex, surgical procedure, tumor volume, alcohol history, smoking history, and the surgeon. The clinical parameters of the patients were obtained from their medical records. The tumor volume was measured using the following formula: tumor volume = 0.5 × length × width^2^.

### Statistical analysis

Data were analyzed using SPSS (version 17.0; SPSS, Chicago, IL, USA). The significance of the differences between groups was assessed using the chi-square test, t-test, Fisher’s exact test, or the nonparametric Mann–Whitney U test, depending on the type of data and distribution. Logistic regression analysis was performed for salivary fistulas. All tests were two-sided, and *statistical significance was set at p* < 0.05.

## Results

Of the 177 patients, 77 were female and 100 were male. The mean age of the patients was 46.26 ± 16.46 years. Of these patients, 94 (53.1%) underwent a partial superficial parotidectomy with a branched facial nerve dissection, 75 (42.4%) underwent a partial superficial parotidectomy with a main trunk facial nerve dissection, and eight (4.5%) underwent a total parotidectomy with a main trunk facial nerve dissection. The most common histological types were pleomorphic adenomas (63/177, 35.6%) and Warthin tumors (54/177, 30.5%).

The clinical parameters are shown in Table 1, and there were no significant differences between the experimental and control groups. The postoperative characteristics are shown in Table 2. The drainage output of the experimental group was significantly lower than that of the control group (*p* = 0.04). The duration of dressing pressure in the experimental group was significantly shorter than that in the control group (*p* = 0.0003). Moreover, the incidence of salivary fistula in the experimental group (9.8%, 8/82) was notably lower than that in the control group (34.7%, 33/95) (*p* < 0.0001). In the logistic regression model for salivary fistula development, both the use of bipolar coagulation forceps (*p* = 0.0021) and drainage output (*p* = 0.0237) were associated with the presence of salivary fistula (Table 3). Hence, this study revealed that bipolar coagulation forceps can be used in a parotidectomy to reduce the incidence of salivary fistulas.

**Table 1:** Clinical characteristics of patients treated with or without bipolar coagulation forceps

Group	No. of patients (%)		*p* value
	With bipolar coagulation forceps (n = 82)	Without bipolar coagulation forceps (n = 95)	
Age (y)	46.76 ± 16.01	45.66 ± 17.04	0.6590
Sex			0.3647
Man	43 (53.7)	57 (60)	
Woman	38 (46.3)	38 (40)	
Surgical procedure			0.4377
A	40 (48.8)	54 (56.8)	
B	37 (45.1)	38 (40)	
C	5 (6.1)	3 (3.2)	
Pathology			0.6983
Pleomorphic adenoma	30 (36.6)	33 (34.7)	
Warthin tumor	23 (28.1)	31 (32.6)	
Branchial cyst	0	1 (1)	
Basal cell adenoma	4 (5)	6 (6.3)	
Monomorphic adenoma	2 (2.4)	2 (2.1)	
Tuberculosis	0	1 (1)	
Hemangioma	1 (1.2)	2 (2.1)	
Benign lymphoepithelial lesion	4 (5)	5 (5.3)	
Lymphoma	0	1 (1)	
Squamous cell carcinoma	3 (3.7)	2 (2.1)	
Chronic sialadenitis	2 (2.4)	2 (2.1)	
Neuroendocrine carcinoma	0	1 (1)	
Oncocytic carcinoma	1 (1.2)	1 (1)	
Neurilemmoma	1 (1.2)	0	
Myoepithelioma	1 (1.2)	0	
Oxyphilic adenoma	0	0	
Ductal papilloma	1 (1.2)	0	
Cystadenoma	2 (2.4)	1 (1)	
Mucoepidermoid carcinoma	1 (1.2)	2 (2.1)	
Acinic cell carcinoma	5 (6.1)	2 (2.1)	
Adenoid cystic carcinoma	0	1 (1)	
Lipoma	0	1 (1)	
Salivary duct carcinoma	1 (1.2)	0	
Tumor volume	14.53 ± 19.05	14.35 ± 34.37	0.9681
Alcohol history			0.2402
Yes	43 (52.4)	42 (44.2)	
No	39 (47.6)	53 (55.8)	0.8914
Smoking history			
Yes	38 (46.3)	45 (47.4)	
No	44 (53.6)	50 (52.6)	0.4507
Surgeons			
a	46(56.1)	48 (49.5)	
b	36 (43.9)	47 (50.5)	

The data showed as mean ± SD; Surgical procedure, A, tumor and partial superficial parotidectomy with branched facial nerve dissection; B, tumor and partial superficial parotidectomy with main trunk facial nerve dissection; C, tumor and total parotidectomy with main trunk facial nerve dissection; Surgeons, a and b represents the two senior surgeons

**Table 2:** The postoperative characteristics of patients treated with or without bipolar coagulation forceps

Group	No. of patients (%)		*p* value
	With bipolar coagulation forceps (n = 82)	Without bipolar coagulation forceps (n = 95)	
Draining output (ml)	80.72 ± 57.08	118.9 ± 158.5	0.0400
Duration of pressure dressing application (day)	3.488 ± 1.259	4.347 ± 1.724	0.0003
**Salivary fistula**			< 0.0001
Yes	8 (10)	33 (34.7)	
No	74 (90)	62 (65.3)	

The data showed as mean ± SD

**Table 3:** Logistical regression analyses of Salivary fistula and clinical characteristics

Group	Regression coefficient	OR (95% CI)	*p* value
Age	0.0007150	− 0.003081 to 0.004511	0.7105
Sex	0.06795	0.07266 to 0.2086	0.3414
Surgical procedure	0.03353	− 0.1379 to 0.07090	0.5270
Bipolar coagulation forceps	0.1929	− 0.3150 to − 0.07093	0.0021
Tumor volume	0.0008940	0.001243 to 0.003031	0.4100
Alcohol history	0.07631	0.09550 to 0.2481	0.3818
Smoking history	0.05051	− 0.2380 to 0.1370	0.5956
Draining output	0.0006957	0.00009396 to 0.001297	0.0237
Surgeons	0.08133	0.2044 to 0.04176	0.1939
Duration of pressure dressing application	0.03716	− 0.01080 to 0.08512	0.1280

CI, confidence interval; OR, odds ratio

## Discussion

Salivary fistula is a relatively common complication in patients who have undergone a parotidectomy. They can persist for a long period even after complete wound healing, which may lead to visible scarring and wound infection. To prevent these issues, we aimed to investigate the effects of bipolar coagulation forceps use on salivary fistulas. In this study, the results suggested that parotidectomy patients treated with bipolar coagulation forceps had a lower incidence of salivary fistulas.

The confounding factors in this study, including age, sex, alcohol history, and smoking history, served as general clinical parameters, which may have a potential effect on the incidence of salivary fistulas. Additionally, other confounding factors, including surgical procedure, tumor volume, and the surgeon could determine the manner of the surgical procedures and prognosis [7]. In the logistic regression model for salivary fistula development, drainage output was associated with the presence of salivary fistula, which was consistent with previous studies [3, 9].

Disease management consists of diagnosis and therapy. A recent study suggested that drain fluid amylase served as a predictor of postoperative salivary fistula in benign parotid tumors [3]. Parotid capsule persistence was correlated with the presence of salivary fistula [3], while closure of the parotid capsule had no effect on the salivary fistula postoperatively [10]. The results indicated that the size of the parotidectomy wound area determined the occurrence of salivary fistula postoperatively.

To decrease the incidence of salivary fistula, previous studies have proposed many therapeutic techniques, including a reduction in oral intake and parenteral feeding [11], sewing the site of the salivary leak [10], a restraining bandage [3], use of anticholinergic agents [12, 13], injection of botulinum toxin [14–16], application of cyanoacrylates after closing the skin incision [17], resection of the tympanic nerve [18], and radiation therapy [19]. In the present study, our results showed that the use of bipolar coagulation forceps decreased the incidence of salivary fistula in patients who had undergone a parotidectomy. It can serve as a novel treatment for salivary fistulas. As expected, patients in the control group had a larger drainage output volume than those in the experimental group.

Electrosurgical instruments that produce heat have been used to control bleeding during surgical procedures [20]. Bipolar coagulation forceps are always used as hemostatic devices during an operation [21, 22]. In the present study, we found that patients treated with bipolar coagulation forceps had a lower incidence of salivary fistula. Due to the fragile characteristics of the parotid gland, when the parotid wound region is treated with surgical sutures, wound dehiscence easily develops postoperatively, causing a salivary fistula. However, bipolar coagulation forceps can be used to seal the parotid wound region to facilitate fresh gland wound healing. This may explain the high incidence of salivary fistula in the parotid wound-treated surgical suture group. In a study by Zou, methylene was injected into the Stensen’s duct to ligate the broken duct [23], while the broken intercalated duct and secretory duct were not ligated. The use of bipolar coagulation forceps can resolve this problem. Convenience is the greatest advantage of bipolar coagulation forceps and is commonly used for hemostasis.

## Conclusions

In this study, we found that the use of bipolar coagulation forceps decreased the incidence of salivary fistulas in patients who had undergone a parotidectomy. The use of bipolar coagulation forceps is a safe, effective, and convenient method to prevent salivary fistulas in patients who undergo a parotidectomy.

## Data Availability

The datasets used and/or analysed during the current study are available from the corresponding author on reasonable request.

## Abbreviations

CI: Confidence interval
OR: Odds ratio

## References

[1] Gandolfi MM, Slattery W (2016). Parotid gland tumors and the facial nerve. Otolaryngol Clin North Am.

[2] Maahs GS, Oppermann Pde O, Maahs LG, Machado Filho G, Ronchi AD (2015). Parotid gland tumors: a retrospective study of 154 patients. Braz J Otorhinolaryngol.

[3] Lu Y, Zhang S, Peng C, Yang W, Zhang C, Ren Z (2020). Drain fluid amylase as a predictor of postoperative salivary fistula in cases with benign parotid tumours. BMC Oral Health.

[4] Brill AI (2008). Bipolar electrosurgery: convention and innovation. Clin Obstet Gynecol.

[5] Taheri A, Mansoori P, Sandoval LF, Feldman SR, Pearce D, Williford PM. Electrosurgery: part II. Technology, applications, and safety of electrosurgical devices. J Am Acad Dermatol. 2014;70(4):607.e601–607.e612. 10.1016/j.jaad.2013.09.055 24629362

[6] Malis LI. Electrosurgery and bipolar technology. Neurosurgery. 2006;58(1 Suppl):ONS1–12; discussion ONS11–12. 10.1227/01.neu.0000204216.05933.19 16479623

[7] Britt CJ, Stein AP, Gessert T, Pflum Z, Saha S, Hartig GK (2017). Factors influencing sialocele or salivary fistula formation postparotidectomy. Head Neck.

[8] Wu K, Lei JS, Mao YY, Cao W, Wu HJ, Ren ZH (2018). Prediction of flap compromise by preoperative coagulation parameters in head and neck cancer patients. J Oral Maxillofac Surg.

[9] Jiang J, Jia MY, Cai Z, Yuan RT, Wang K, Zhang K, Bu LX (2014). The effect evaluation of suction drainage to prevent fistula after superficial parotidectomy. Shanghai kou qiang yi xue = Shanghai J Stomatol.

[10] Mantsopoulos K, Goncalves M, Iro H (2018). Transdermal scopolamine for the prevention of a salivary fistula after parotidectomy. Br J Oral Maxillofac Surg.

[11] Marchese-Ragona R, De Filippis C, Marioni G, Staffieri A (2005). Treatment of complications of parotid gland surgery. Acta otorhinolaryngologica Italica : organo ufficiale della Societa italiana di otorinolaringologia e chirurgia cervico-facciale.

[12] Becelli R, Morello R, Renzi G, Matarazzo G (2014). Use of scopolamine patches in patients treated with parotidectomy. J Craniofac Surg.

[13] Gallo A, Manciocco V, Pagliuca G, Martellucci S, de Vincentiis M (2013). Transdermal scopolamine in the management of postparotidectomy salivary fistula. Ear Nose Throat J.

[14] Costan VV, Dabija MG, Ciofu ML, Sulea D, Popescu E, Boisteanu O (2019). A functional approach to posttraumatic salivary fistula treatment: the use of botulinum toxin. J Craniofac Surg.

[15] Graillon N, Le Roux MK, Chossegros C, Haen P, Lutz JC, Foletti JM (2019). Botulinum toxin for ductal stenosis and fistulas of the main salivary glands. Int J Oral Maxillofac Surg.

[16] Send T, Bertlich M, Eichhorn KW, Bootz F, Jakob M (2019). Management and follow-up results of salivary fistulas treated with botulinum toxin. Laryngoscope.

[17] Marcus AJ, Nasser NA (1998). Case report: The treatment of a chronic parotid cutaneous fistula by the injection of a solution of lipiodol with cyanoacrylate. Clin Radiol.

[18] Davis WE, Holt GR, Templer JW (1977). Parotid fistula and tympanic neurectomy. Am J Surg.

[19] Christiansen H, Wolff HA, Knauth J, Hille A, Vorwerk H, Engelke C, Rödel R, Laskawi R (2009). Radiotherapy : an option for refractory salivary fistulas. HNO.

[20] Vellimana AK, Sciubba DM, Noggle JC, Jallo GI. Current technological advances of bipolar coagulation. Neurosurgery. 2009;64(3 Suppl):ons11–18; discussion ons19. 10.1227/01.NEU.0000335644.57481.97 19240559

[21] Manouras A, Markogiannakis HE, Kekis PB, Lagoudianakis EE, Fleming B (2008). Novel hemostatic devices in thyroid surgery: electrothermal bipolar vessel sealing system and harmonic scalpel. Expert Rev Med Devices.

[22] Mikami T, Wanibuchi M, Mikuni N (2012). Bumping phenomenon during continuous coagulation with bipolar forceps. Neurol Med Chir.

[23] Zou HW, Li WG, Huang SY, Chen ZW, Zhang DS (2019). New method to prevent salivary fistula after parotidectomy. Br J Oral Maxillofac Surg.

